# Sf-PHB2, A new transcription factor, Drives WSSV *Ie1* Gene Expression via a 12-bp DNA Element

**DOI:** 10.1186/1743-422X-9-206

**Published:** 2012-09-17

**Authors:** Guoda Ma, Li Yu, Qian Wang, Wei Liu, Yudong Cui, Jimmy Kwang

**Affiliations:** 1 Division of Livestock Infectious Diseases, State Key Laboratory of Veterinary Biotechnology, Harbin Veterinary Research Institute, Chinese Academy of Agricultural Sciences, 427 Maduan Street, Harbin, 150001, P. R. China; 2 College of Life Science & Technology, Heilongjiang Bayi Agricultural University, Daqing, 163319, P. R. China; 3 Animal Health Biotechnology, Temasek Life Sciences Laboratory, the National University of Singapore, Singapore, 117604, Singapore

**Keywords:** WSSV, *ie1* promoter, 12-bp motif, Transcription factor, Sf-PHB2

## Abstract

**Background:**

The WSSV immediate early gene *ie1* is highly expressed throughout viral infection cycle and may play a central role in initiating viral replication during infection.

**Results:**

Here, a detailed characterization of the *ie1* promoter was performed using deletion and mutation analyses to elucidate the role of the individual promoter motifs. Three results were obtained: 1) the *ie1* promoter is a classical eukaryotic promoter that contains the initiator element (Inr) and TATA box responsible for the basal promoter activity; 2) mutation or truncation of a predicted Sp1 site decreased the level of promoter activity by about 3-fold, indicating that the Sp1 site is an important *cis*-element of the promoter; and 3) truncation of a 12-bp sequence that resides at -78/-67 of the *ie1* promoter decreased the level of promoter activity by about 14-fold, indicating that the 12-bp motif is a critical upstream element of the *ie1* promoter for binding of a strong transcription factor to drive the *ie1* gene expression in the cells. Further, the 12-bp DNA binding protein was purified from the nuclear proteins of Sf9 cells using DNA affinity chromatography, and was identified as a homologue of the prohibitin2 protein (named as Sf-PHB2) using mass spectrometry. Furthermore, the DNA binding activity of Sf-PHB2 was verified using a super shift analysis.

**Conclusion:**

These results support that the Sf-PHB2 is a novel transcription factor that drives WSSV *ie1* gene expression by binding to the 12-bp DNA element.

## Background

White spot syndrome (WSS), which first appeared in Southeast Asia at the beginning of the 1990s and spread globally, is the most serious infectious disease of cultured shrimp [1]. White spot syndrome virus (WSSV), the causative agent of WSS, is a large rod-shaped virus with circular double-stranded DNA (dsDNA) that belongs to the new virus family Nimaviridae, genus Whispovirus [2]. A sequence analysis has shown that the WSSV genome encodes more than 180 open reading frames (ORFs), most of which have functions that are still unknown [3-5]. Only 6% of the WSSV ORFs have putative homologues in databases [3], and the evolutionary singularity of this virus makes it difficult to directly apply other viral infection models to interpret the infectious strategy of WSSV. In addition, no continuous cell line exists in which WSSV can be grown, which makes it difficult to study WSSV. To date, more than 40 structural protein genes of the virus have been identified, and other WSSV genes with known functions have been described, including immediate early genes, latency-related genes, ubiquitination-related genes, and anti-apoptosis genes [6-8]. So far, the molecular mechanisms that are involved in the control of WSSV gene transcription and the replication cycle of WSSV are still largely unknown.

As with most of the large dsDNA viruses, such as baculovirus and herpes virus, WSSV genes can be classified as early or late genes based on their temporal expression profiles. These viruses are expressed in a coordinated and cascaded fashion under the control of several different promoters [9,10]. The immediate early gene products are synthesized immediately after viral infection and rely primarily on host factors for their expression. Several immediate early genes encode important transregulators of viral gene expression and replication [10-12]. To date, 20 *ie* genes have been identified in WSSV, of which 4 exhibit transcription activity [10,11]. The WSSV *ie1* gene is highly expressed throughout the WSSV infection cycle [11], the protein encoded by *ie1* (IE1) contains a Cys2/His2-type zinc finger that is a domain involved in DNA-protein interactions. Therefore, the IE1 protein has been reported to act as a transcription factor [13], and the *ie1* gene may play critically important roles in the regulation of WSSV transcription and in the infection cycle of the virus.

We have previously reported on the pan-activity of the strong *ie1* gene promoter in many cells including Sf9 insect cells [14], the cell line Sf9 has been extensively used to study WSSV genes at the cellular level, even though it is not permissive to WSSV infection [14,15]. Based on structural prediction the *ie1* gene promoter has a putative TATA box and a downstream Inr element that is similar to other WSSV early gene promoters [11]. Many dsDNA virus early genes have promoters that resemble the typical RNA polymerase II promoters that are found in insect cells and the cells of other organisms [16], these promoters are readily transcribed in uninfected insect cells, indicating that they utilize cellular factors for transcription activation. In other words, the promoters of these immediate early genes are recognized by host cell factors, and no viral factors are required for this promoter activity.

In the present study, the transcription start site of the *ie1* gene was determined and we found that its promoter contained both early and late elements. By functionally mapping a series of the truncates a new 12-bp regulatory element of the *ie1* promoter was identified in the -78 to -67 bp region that was related to the transcription start site, which was shown to be essential in controlling the high-level expression of this gene. To delineate the transcription regulation mechanism of the 12-bp element of the WSSV *ie1* gene promoter, we further purified the 12-bp DNA binding protein from the Sf9 nuclear extracts using DNA affinity chromatography, and the purified protein was identified as a homologue of the mosquito prohibitin2 using mass spectrometry. The gene encoding the 12-bp binding protein was cloned from Sf9 cells and named *sf-phb2*. The interaction between Sf-PHB2 and the 12-bp motif was further verified using electrophoretic mobility shift assays (EMSA) and an antibody super shift. In addition, a classical nuclear localization signal and a helix-turn-helix domain that was located in Sf-PHB2 provided structural evidence for its role as a novel transcription factor.

These results supported that the Sf-PHB2 protein is a critical transcription factor that drives WSSV *ie1* gene expression by binding to the 12-bp DNA element. These data are important not only to understand of the general mechanisms that control gene expression, but also to provide additional insight into WSSV replication in host cells.

## Results

### Transcription start site of the WSSV *ie1* gene

**Figure 1:**
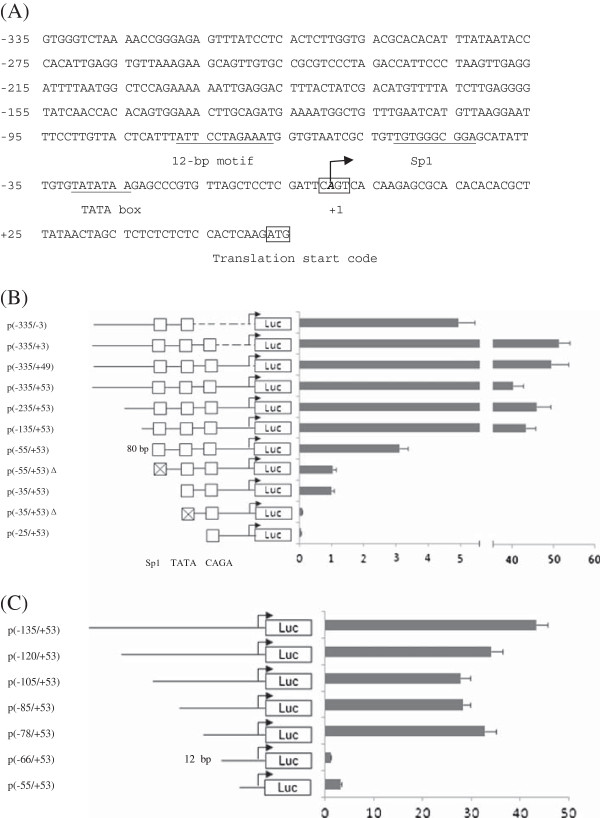
**Functional analysis of the WSSV *ie1 *promoter through deletion and mutation.** (**A**) The 388 bp sequence of the WSSV *ie1* promoter is shown. The numbers are relative to the transcription start site (+1). The specific protein binding sites such as Sp1 and the TATA box are underlined. The transcription start site is shown in italics and bold. (**B**) The schematic depicts the features of the WSSV *ie1* promoter that is positioned between the putative translational start site and its 388-bp upstream sequence. A series of truncated promoter fragments were constructed in front of the luciferase gene in a promoter-less vector phRG-B. For the 5' end and the 3' ends, each deletion and mutation of the promoter is depicted and named as shown in the figure. The putative Sp1-binding site and TATA box were mutated [p(-55/+53Δ)and p(-35/+53Δ)] using PCR with a mutated primer. (**C**) Functional mapping of the *cis*-elements within the 80-bp sequence (from -135 to -55 bp). The region spanning 80 bp of the promoter was progressively deleted from its 5'-end.

To determine the transcription start site of the WSSV *ie1* gene, 5'-RACE was performed using the RNA that was isolated from the WSSV infected shrimps with the *ie1* gene specific primers. A DNA fragment resulting from the 5'-RACE nested was cloned and sequenced. The sequence analysis revealed that in 6 of the 10 selected clones, the transcription start site was located 24 bp downstream of a putative TATA box (TATATAAG) and 53 bp upstream of the predicted ATG translation start codon. In the other 4 clones, the 5' termini were 52 or 51 bp upstream of the putative ATG. These data indicated that the transcription start point of the *ie1* gene initiated from the adenine within the CAGT motif, 53 bp upstream of the ATG codon (Figure 1A).

### Core promoter of the WSSV *ie1* gene

The sequence analysis of the WSSV *ie1* promoter demonstrated the presence of several putative transcription factor binding sites, which could be important for promoter activity (Figure 1A). To detect the activity of the WSSV *ie1* promoter in the Sf9 cells, a PCR amplified 388 bp sequence (from -335 to +53 bp) was cloned into the phRG-B luciferase reporter vector. A series of deletions and mutations from the 5' side of the *ie1* promoter were generated to determine the minimal sequence that was required for the basal level of promoter activity (Figure 1B). The results showed that the shortest construct p(-25/+53) that contained the CAGT motif as a putative initiator element (Inr) retained only a minimal level of promoter activity and that the addition of a TATA box p(-35/+53) resulted in a 17.8-fold increase in the activity level. At the same time, a mutation of the TATA box (p(-35/+53)Δ) caused a 10.5-fold decrease in the level of activity. To further assess the function of the CAGT as a possible Inr, 3' deletions from the *ie1* promoter were constructed by truncating CAAG (p(-335/+49)), 50 bp upstream of the translational start site (p(-335/+3)) and Inr consensus sequence TCAGTC (p(-335/-3)). The results revealed that a deletion of the Inr consensus sequence caused a 10-fold decrease in the level of activity compared to the wild type promoter p(-335/+53), whereas deletions that were downstream of the Inr had no obvious effect on the level of promoter activity (Figure 1B). This finding indicated that the sequence TCAGTC is an initiator element that constitutes a core promoter of the *ie1* together with the TATA box.

### Proximal promoter element of the WSSV *ie1* gene

The deletion of an 80-bp subsequence, from -135 to -55 bp, caused an 11-fold decrease in the level of promoter activity, and a mutation (p(-55/+53) Δ) or deletion p(-35/+53) of the potential Sp1-binding motif caused a 2.6-fold or 2.8-fold decrease in the level of promoter activity, respectively, indicating that the Sp1-binding motif and the 80 bp sequence are required for maximal transcription that is driven by the *ie1* promoter. In contrast, the deletion of putative AP-1/CRE and the My and Myc motifs p(-235/+53) or their proximal downstream p(-135/+53) had no significant effect on the level of promoter activity (Figure 1B).

### A 12-bp motif is a new activating promoter element that is responsible for the main activity of the WSSV *ie1* promoter

Our observation that the 80-bp region is the main upstream sequence that is responsible for the *ie1* promoter activity suggested the presence of a new activating promoter element(s) in this 80-bp region. Therefore, we searched for activating element(s) by performing further deletions. The results indicated that the luciferase activity dramatically dropped 14-fold from p(-78/+53) to p(-66/+53) (Figure 1C). The difference between these two deletions is a 12-nt fragment that is an imperfect inverted repeat, 5'-^-78^ATTTATTCCTAG^-67^-3', indicating that the 12-bp sequence is the critical upstream element of the WSSV *ie1* promoter. Importantly, the 12-bp DNA element is a new activating promoter element that does not contain the previously identified binding sites recognized by known cellular transcription factors. Thus, we proposed that there is an unknown transcription factor that regulates WSSV *ie1* gene expression via binding the 12-bp DNA motif.

### The protein binding to the 12-bp DNA is prohibitin 2 from Sf9 cells

**Figure 2:**
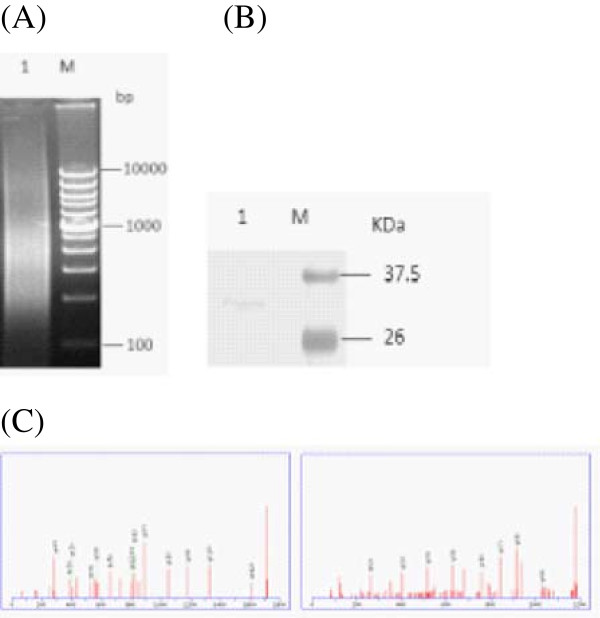
**Purification of the 12-p DNA-binding protein using a tandem DNA-affinity columns.** (**A**) Concatamerization of specific DNA oligonucleotides using self-priming PCR. PCR products with the concatamers of the 12-bp oligonucleotide were analyzed using agarose electrophoresis. 1, self-primer PCR products; M, DNA ladder. (**B**) SDS-PAGE analysis of the purified 12-bp DNA-binding protein. 1, the purified protein; M, protein markers. (**C**) Identification of the captured proteins using mass spectrometry, two representative peptide mass fingerprint profiles VPWFQYPIIYDIR (**A**) and FNASQLITQR (**B**).

To identify the protein(s) that binds the 12-bp DNA fragment, the nuclear proteins from the Sf9 cells were purified by binding the biotinylated DNA and using streptavidin affinity chromatography. In SDS-PAGE analysis, the affinity-purified proteins revealed a band of 32 kDa after sliver staining (Figure 2B). The protein was excised from the gel and was digested with trypsin *in situ*. Further, the tropic peptides were extracted from the gel slices for analysis using mass spectrometry. Two peptide sequences, VPWFQYPIIYDIR and FNASQLITQR, which corresponded to the tropic peptide were identified (Figure 2C) to be matched the mosquito prohibitin2 (PHB2) proteins (GenBank accession number XM_001842599) in the non-redundant database, indicating that the affinity-purified 32 kDa protein is a homologue of PHB2 of *Spodoptera frugiperda*. To date, no reports have documented cloning of the gene encoding PHB2 from Sf9 cells.

### *In silicon* cloning and evolutionary analysis of the Sf-PHB2 gene

It is a common strategy to clone protein family cDNA using degenerate primer PCR. Using one pair of the primers, a partial cDNA of the PHB2 gene was obtained from the Sf9 cells using RT-PCR. To obtain full-length cDNAs of the PHB2 gene from the Sf9 cells, we used this sequence as a query to blast against the *Spodoptera frugiperda* EST database. As a result, a contig was assembled on the basis of several homologous ESTs (DY897934, DY793476, DV076437 and DY784502). Furthermore, we predicted the open reading frame of the contig and confirmed the complete cDNA of the gene using BLAST. We nominated the novel gene as *sf-phb2* (GenBank accession number HQ337624).

The full length of the *sf-phb2* gene is 1297 bp, including 112 nucleotides in the 5' -UTR 285 nucleotides in the 3'-UTR, and an ORF that encodes 299 amino acids, with a predicted mass of 32 KDa. The sequence analysis revealed that the Sf-PHB2 protein contains a PHB domain at residues 41-202, a nuclear localization sequence (NLS) at residues 87-90 and a helix-turn-helix (HTH) domain at residues 136-181.

**Figure 3:**
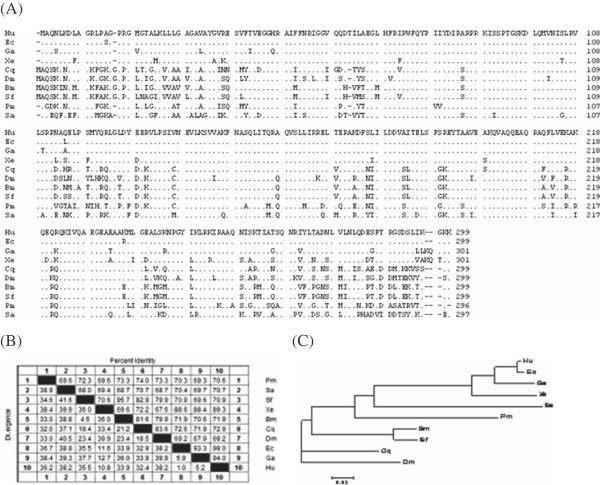
**Evolutional analysis of the prohibitin 2 gene family.** (**A**) Multiple sequence alignment of the deduced amino acid sequence of the Sf-PHB2 gene with 9 ancient PHB2 family members. These sequences are available from the GenBank under the accessions: Dm = *Drosophila melanogaster* (NP_725832.2), Sf = *Spodoptera frugiperda* (HQ337624), Pm = *Penaeus monodon* (ACD13589.1), Hu = *Homo sapiens* (NP_009204.1), Cq = *Culex quinquefasciatus* (EDS26618.1), Nv = *Nematostella vectensis* (XP_001634411.1), Ga = *Gallus gallus* (NP_001074354.1), Bm = *Bombyx mori L* (NP_001040326.1), Ec = *Equus caballus* (XP_001497915.1), Xe = *Xenopus* (NP_001016551.1). (**B**) Sequence homology of the prohibitin2. The matrix of the percentage similarities of the amino acids for the 10 members of the PHB2 family. (**C**) Phylogenetic analysis of the PHB2 protein family members.

Blastp searches were performed using the amino acid sequence of the Sf-PHB2 from the NCBI database, and the obtained homologues of the Sf-PHB2 are listed in Figure 3. Multiple sequence alignments were performed using MEGA4. To investigate the evolutionary relatedness between *Spodoptera frugiperda* PHB2 and other PHB2s, the 10 homologous sequences were subjected to a phylogenetic analysis and the resulting identity matrix is shown in Figure 3 B and C. The results revealed that Sf-PHB2 is relatively close to other insect PHB2s, exhibiting 95.7%, 82.9% and 79.9% amino acid sequence similarity to other insect *Bombyx mori* L, *Culex quinquefasciatus* and *Drosophila melanogaster,* respectively. However,Sf-PHB2 shares only 70.9% of its sequence with its human counterpart.

### Sf-PHB2 protein is localized in the cytoplasm and the nuclei of Sf9 cells

**Figure 4:**
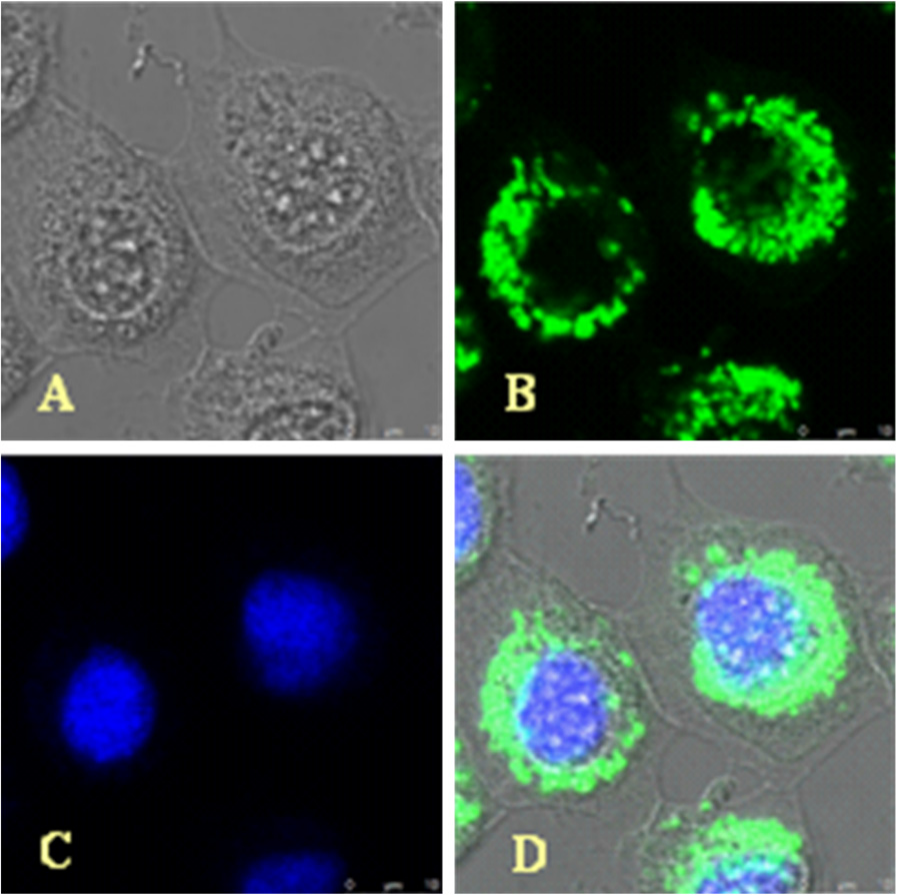
**Intracellular localizations of the Sf-PHB2 in the Sf9 cells.** (**A**) Normal Sf9 cells. (**B**) The Sf-PHB2 was visualized using mouse antibody against Sf-PHB2 and FITC-conjugated goat anti-mouse IgG antibody. (**C**) The nuclei were visualized by counterstaining with DAPI. (**D**) Merged FITC and DAPI signals. Original magnification: 400 x. bar = 1 μm.

Because Sf-PHB2 was purified from the nuclear exacts and contains putative nuclear localization signals, we investigated whether Sf-PHB2 is indeed a nuclear protein. To determine the subcellular distribution pattern of the Sf-PHB2 protein, indirect immunofluorescence staining using Sf-PHB2 antibody was performed in the Sf9 cells. As shown in Figure 4, more Sf-PHB2 protein was present in the cytoplasm than in the nucleolus of the Sf9 cells. No fluorescent staining was observed in the Sf9 cells using pre-immune serum (data not shown). These results indicated that Sf-PHB2 is a nuclear protein in the Sf9 cells. The nuclear localization of Sf-PHB2 supports its potential role in the regulation of *ie1* gene expression.

### Sf-PHB2 binds directly to the 12-bp *cis*-acting element of the WSSV *ie1* promoter

**Figure 5:**
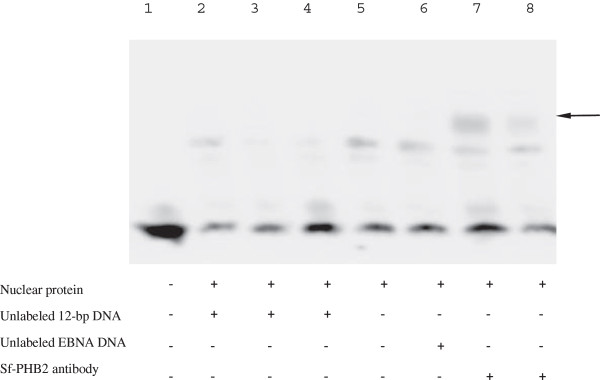
**Sf-PHB2 specifically binds to the 12-bp sequence of the WSSV *ie1 *promoter.** The biotin-labeled probe was incubated in the absence (lane 1) or presence (lanes 2-8) of the nuclear extracts from the Sf9 cells. For the competition experiments, an equal amount of unlabelled EBNA oligos (lane 6) or 2-, 5- and 10-fold molar excesses of the unlabelled probe (lanes 2, 3 and 4) were added to the binding reaction. Lanes 7 and 8, the anti-PHB2 antibody-mediated supershift experiment. The arrow indicates the supershift.

As described above, we purified the Sf-PHB2 protein from the Sf9 cells using DNA affinity chromatography. To further investigate the interaction between the Sf-PHB2 protein and the 12-bp DNA, the affinity of the Sf-PHB2 to the 12-bp sequence was evaluated using an EMSA analysis with nuclear extracts that were prepared from the Sf9 cells. For the super shift assay, a polyclonal antibody was generated against the Sf-PHB2 protein, and the results are shown in Figure 5. If the nuclear extracts were used alone, a protein-DNA complex was detected (lane 5). This complex appeared to be specific because the addition of the unlabeled the 12-bp probe prevented its formation (lanes 2, 3 and 4), whereas an unlabeled EBNA probe did not prevent its formation (lane 6). A super shift analysis was then performed to identify the protein that was bound to the 12-bp element. The protein-DNA complex band was super shifted by the addition of 1 or 0.5 μg Sf-PHB2 antibody (lane 7 and 8). Conversely, similar amounts of anti-IgG control antibodies had no significant effect (data not shown). These results showed that the Sf-PHB2 binds specifically to the 12-bp *cis*-acting element of the WSSV *ie1* promoter.

Taken together, during the functional mapping of the WSSV *ie1* gene promoter, we found a new 12-bp regulatory element that is responsible for most of the promoter activity. Furthermore, we purified the 12-bp DNA binding protein using DNA affinity chromatography and identified it as a homologue of the mosquito prohibitin2 using mass spectrometry. Lastly, we verified the DNA-protein interaction between Sf-PHB2 and the 12-bp motif using EMSA and an antibody super shift. In addition, Sf-PHB2, which carried a classical nuclear localization signal and a DNA binding domain HTH, was localized to the nucleolus of the Sf9 cells. These results support that the Sf-PHB2 is a novel transcription factor that drives WSSV *ie1* gene expression by binding to the 12-bp DNA element.

## Discussion

**Figure 6:**
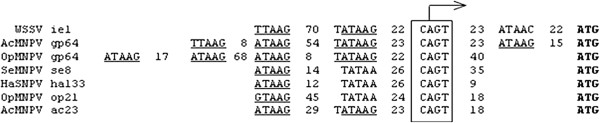
**Comparison of the early and late promoter subsequences between the WSSV *ie1 *gene and the insect baculovirus genes.** The numbers indicate the number of nucleotides between the regulatory elements shown. ATG is the translation start of the coding sequence. The underlined sequences are the late promoter sequences. The boxed regions are the early promoter sequences, and the arrow is an early mRNA start site.

The WSSV *ie1* gene contains a strong, pan-activity promoter and is highly expressed throughout the infection cycle [11,14]. Expression patterns have been shown to be closely related to promoter structure. In the present study, the transcription start site of the *ie1* gene was determined using the 5' RACE method at 53 nt upstream of the translation start codon, the transcription of which is initiated at a conserved CAGT subsequence, a motif that is a feature of early genes in many insect baculoviruses [17]. *Liu* et al. reported that the transcription start site of the *ie1* gene was localized 52 nt upstream of the ATG codon [11], which leads to an *ie1* transcript that is one nucleotide less than that determined here. In addition, as shown in Figure 6, two late promoter motifs (T/ATAAG) exist at 22 or 98 bp upstream of the *ie1* transcription start site, which is similar to the arrangement seen in the insect baculovirus. [18] In all insect baculovirus genomes that have been sequenced, a combination of both early and late promoter elements have been identified upstream of several genes. This arrangement facilitates gene expression both before and after DNA replication. In the baculoviruses AcMNPV and OpMNPV, approximately 15% of the predicted reading frames have both early and late promoter elements within 120 bp of the translation initiation codon [19,20]. For the WSSV *ie1* gene, the transcript was present throughout the virus infection cycle [10], and the presence of both early and late promoter elements in the 5′ regulatory region suggests that it uses the early promoter early in infection and shifts to the late promoter at later time points. The late expression of this protein would allow for the control of the level of WSSV replication throughout its infection cycle. Transcription machinery is highly conserved, and the basic mechanism of transcription is remarkably conserved across species, even in bacteria and eukaryotes. Because WSSV and some insect baculoviruses share the unique feature of promoter structure and function, we suggest that they have a common ancestor. However, they may have diverged during an ancient era, causing their genes to share little or no sequence homology.

The identification of functional *cis*-acting DNA regulatory elements is a critical step towards understanding gene expression regulation. In the present study, we cloned 388 bp of the 5' upstream promoter of the *ie1* gene and analyzed the promoter activity through a deletion and mutation with a luciferase reporter. In the deletion studies, the -35 to +3 region that contained the transcription starting sequence CAGT and a putative TATA box was sufficient for basal promoter activity, whereas the fragment from -235 to +53 exhibited the highest level of promoter activity. The sequence analysis of the 388 bp fragment revealed a stretch of several adjacent putative transcription factor binding sites, of which only the Sp1 binding site was functional. The critical *cis*-acting sequence of the WSSV *ie1* promoter is a 12-bp imperfect inverted repeat, 5'-^-78^ATTTATTCCTAG^-67^-3', which is predicted to bind an unknown transcription factor that is very important for driving the expression of the *ie1* gene. In summary, the core promoter of the *ie1* gene consists of a TATA box and the transcription initiating element CAGT, and a tentative Sp1 binding site and the 12-bp imperfect inverted repeat were essential for maximal promoter activity. The transcription start site of the *ie1* gene is located within the transcription initiating element CAGT.

Thereafter, the 12-bp DNA sequence of the *ie1* gene was used as the affinity material to capture the transcription factor(s) that recognizes this specific DNA sequence from the nuclear proteins of the Sf9 cells, and only Sf-PHB2 was identified as the protein to bind the 12-bp DNA.

Although very important advances in the understanding of the PHB2 have been made in the decade since its discovery, the functions of PHB2 are studied in vertebrates and only partially understood [21-25]. In addition to its role as a chaperone protein in mitochondria, compelling evidence now exists that indicated that it is localized in the nucleus and can modulate transcription activity by interacting with various transcription factors, including the steroid hormone receptors, either directly or indirectly [25-27]. To date, there have been no reports of PHB2 acting as a transcription factor that functions by binding the DNA of the promoter.

In the present study, we cloned sf-*phb2* gene and proposed that Sf-PHB2 acts as a transcription factor to modulate the expression of the WSSV *ie1* gene via a 12-bp DNA element in Sf9 cells. In addition, we concluded that this proposed idea is correct. This conclusion rests on four observations. First, DNA affinity chromatography is a powerful method for purifying DNA-binding proteins. Several transcription factors such as the lac repressor protein [28], Sp1 [29] and C/EBP [30], have been purified using DNA affinity chromatography. In this study, we captured the Sf-PHB2 protein from the Sf9 nucleolus using this classical method. Second, we have demonstrated using gel mobility shift competition assays and an antibody super shift that Sf-PHB2 directly binds to the 12-bp motif of the WSSV *ie1* promoter. Third, the NLS of Sf-PHB2 and its expression in the nucleolus of the Sf9 cells support the hypothesis that Sf-PHB2 may play a role as a transcription factor in the regulation of *ie1* gene expression. Lastly and most notably, there is a putative helix-turn-helix (HTH) domain at the C-terminal region of Sf-PHB2, which is a typical DNA-binding domain [31], providing structural evidence that Sf-PHB2 may act as a transcription factor. The HTH motif also exists in the PHB2 proteins of mosquitoes, moths and other invertebrates but does not exist in the PHB2 proteins of vertebrates. Shrimp, as an invertebrate, its PHB2 protein contains an HTH DNA binding domain and nuclear localization signal; furthermore, it has been shown that the PHB2 is expressed differentially between WSSV-infected tissue and normal tissue in shrimp [32]. In addition, only one protein was got from the DNA affinity chromatography, which excluded the possibility of Sf-PHB2 as a transcription regulator. These results together with those of our studies, strongly support that PHB2 is also used as a transcription factor to regulate the gene expression of *ie1* and the replication of WSSV in shrimp.

It is very interesting that Sf-PHB2 can directly bind to the *cis*-acting element of the promoter, whereas its homologous proteins in vertebrates play a role in gene expression regulation through the interaction with transcription factors [27] instead of with DNA. Emerging evidence suggests that organism complexity arises from progressively more elaborate regulation of gene expression [33,34]. It has been reported that many new transcription factors/cofactors may have emerged with evolution [35]. Therefore we speculate that PHB2 exercise certain biological function as a transcription factor in invertebrates, whereas it may cooperate with other transcription factors to perform a similar function in vertebrates.

In contrast to our results, Liu et al. reported that signal transducer and activator of transcription (STAT) enhances WSSV *ie1* gene transcription through the *cis*-acting motif (ATTCCTAGAAA) [15,36]. The STAT binding motif has an 8-nt (ATTCCTAG) overlap with the 12-bp sequence that is recognized by the transcription factor Sf-PHB2, which was mapped in the present study.

In summary, the present study has demonstrated for the first time that the WSSV *ie1* promoter has early and late elements that can interpret *ie1* expression throughout the infection cycle. Our data proved that Sf-PHB2 is a new transcription factor that drives *ie1* expression and results in high level of activity of the *ie1* promoter in Sf9 cells. These findings provide insight into transcription regulation and will be helpful in understanding of the life cycle and molecular pathogenesis of WSSV. In the future we will focus on mapping the corresponding functional domains of Sf-PHB2 as a transcription factor, including the transactivation domain and the DNA binding domain. Furthermore, we will confirm that shrimp PHB2 functions as a transcription factor to drive WSSV *ie1* gene expression by binding to the 12-bp DNA element.

## Conclusion

Sf-PHB2 is a novel transcription factor that drives WSSV *ie1* gene expression by binding to the 12-bp DNA element.

## Materials and methods

### Sf9 cell culture

The Sf9 cell line (Invitrogen) from the *Spodoptera frugiperda* pupal ovarian tissue was propagated and was maintained at 28°C in serum-free medium (Sf-900 II SFM, GIBCO BRL) that was supplemented with 50 μg/ml gentamicin.

### Virus, shrimp and artificial infection characteristics

The WSSV infected the healthy subadult *P. monodon* shrimps (15-20 g) was confirmed using two-step PCR, as described previously [37]. The virus was isolated and prepared from the infected shrimp, as described previously [38], with a slight modification. Briefly, WSSV-infected shrimp were homogenized in the presence of liquid nitrogen. The homogenate was suspended in PBS (pH 7.4) and was frozen and thawed three times, which was followed by centrifugation at 4,500 × *g* for 30 min. The supernatant was filtered through a 0.45 μm filter for experimental infection or for the extraction of viral DNA.

For the 5'-RACE analysis, the healthy sub adult *P. monodon* shrimps were inoculated with WSSV as described previously [39]. After 48 h post infection, 5 of the infected shrimp were selected at random, and their heads were excised. The collected samples were immediately frozen and were stored at –80°C.

### Determination of the 5' terminal regions of the *ie1* gene transcript

The 5' region of the *ie1* transcript was identified using rapid amplification of the cDNA 5' ends (5' RACE) with a 5'-RACE kit (Roche), according to the manufacturer's instructions. The total RNA was prepared from the heads of the WSSV-infected shrimp at 6 h post infection. The appropriate gene specific reverse primer R1 (5'-TACAAAGAATCCAGAAATCTCAT-3') was then used for the cDNA synthesis. Before being subjected to PCR, a poly (A) tail was added to the 5' end of the cDNA product using terminal transferase in the presence of dATP. The first round of PCR was performed using the R2 primer (5'-CAAATCAGAATGACCCACTCCATG-3') and an oligodT anchor. The PCR product from the first round was used as the template for the second round of PCR, using the R3 primer (5'-GTACATCCATATGGATGCCGCATT-3') and the anchor primer from the 5' RACE kit. The final product was sequenced, and the resulting sequences were compared with the WSSV genomic sequence using the DNA Strider^TM^1.3.

### Construction of the report plasmids and cell transfection parameters

The 388 bp (from-335 to +53 bp in relation to the transcription start site) fragment of WSSV *ie1* promoter was generated using PCR with the Primer Star high-fidelity Taq DNA polymerase (Takara) and the WSSV genomic DNA as a template. This approach used a forward primer from the 5'-upsteam region of the genomic sequence with a *Kpn*I site and a reverse primer from the downstream region with a *Hin*dIII site. This fragment was cloned into a phRG-B vector. A series of 5' and 3' deletion constructs were generated using PCR with a series of primers with enzyme digestion sites (Table 1).

**Table 1:** **Oligonucleotides used for the generating the deletion mutants and other clones for the WSSV *ie1 *promoter activity assays**

**plasmids**	**primers (5’ → 3’)**
p(-335/+53)	Fwd1 (CGGGTACC GTGGGTCTAAAACCGGGAGA)
	Rev1 (CCAAGCTTCTTGAGTGGAGAGAGAGA)
p(-235/+53)	Fwd2 (CGGGTACC GACCATTCCCTAAGTTGAGGA)/Rev1
p(-135/+53)	Fwd3 (CGGGTACC CTTGCAGATGAAAATGGCTGT)/Rev1
p(-120/+53)	Fwd4 (CGGGTACC GGCTGTTTGAATCATGT)/Rev1
p(-105/+53)	Fwd5 (CGGGTACC GTTAAGGAATTTCCTTGT)/Rev1
p(-78/+53)	Fwd6 (CGGGTACC ATTCCTAGAAATGGTGT)/Rev1
p(-66/+53)	Fwd 7 (CGGGTACC GGTGTAATCGCTGTTGT)/Rev1
p(-35/+53)	Fwd 8 (CGGGTACC TGTGTATATAAGAGCCCGTGT)/Rev1
p(-55/+53)	Fwd 9 (CGGGTACC TGTTGTGGGCGGAGCATATTTGT)/Rev1
p(-335/+49)	Rev2(CCAAGCTT AGTGGAGAGAGAGAGCTAGT)/Fwd1
p(-335/+3)	Rev3(CCAAGCTT ACTGAATCGAGGAGCTAACA)/Fwd1
p(-335/-3)	Rev4 (CCAAGCTT ATCGAGGAGCTAACACGGGCT)/Fwd1
p(-35/+53Δ)	Mut1(TGTTGTGaGaGGAGCATATT)/Rev1/Fwd 8
p(-55/+53Δ)	Mut2(TGTGTATtaAAGAGCCCGTG)/Rev1/Fwd 9

The *Kpn*I and *Hin*dIII sites are underlined.

The Sf9 cells were Transfected in 6-well plates with 1 μg of reporter plasmid. The transient transfection was performed using Effective (Qiagen), according to the manufacturer's instructions. At 48 hours post transfection, the cells were harvested and were assayed for reporter gene activity with the dual-luciferase report assay system (Promega). All transfection were performed in triplicate, and data were analyzed by normalizing firefly luciferase activity to Renilla luciferase activity for each sample. Each construct was tested in three independent transfection.

### Extraction of nuclear proteins from Sf9 cells

For the isolation of the nuclear proteins from the Sf9 cells, NE-PER® nuclear and cytoplasmic extraction reagents (Thermo Scientific) were used. The nuclear proteins were extracted according to the manufacturer's recommendations. The protein concentrations were determined using the Lowry method.

### Affinity purification of the 12-bp DNA binding protein from the Sf9 cells extracts

The DNA binding protein was purified using magnetic affinity particles that were conjugated with the 12-bp DNA fragment. A number of DNA binding sites were generated using a self-priming PCR method with two synthetic direct repeats of complementary single-stranded 5'-end phosphorylated oligonucleotides that included the 12-bp motif. The following forward (F) and reverse (R) phosphorylated single stranded oligonucleotides were used (the 12-bp DNA element is underlined):

5'-phso-ATTCCTAGAAATGGTGTAATCGCATTCCTAGAAATGGTGTAATCGC-3' (F) and 5'- phso- GCGATTACACCATTTCTAGGAATGCGATTACACCATTTCTAGGAAT-3' (R)

The concatenated nucleotides were conjugated with magnetic particles using a DNA-binding protein purification kit (Roche). The DNA binding proteins were purified according to the manufacturer's instructions [40,41]. The collected proteins were separated by SDS-PAGE, and the gel was stained with sliver following protocols from previously published report [42]. The protein bands were excised for protein identification.

### MALDI-TOF MS analysis

The excised protein in-gel was sent to Chinese National Center of Biomedical Analysis (Beijing, China) for protein identification. Briefly, the protein was digested with trypsin (MS-grade, Sigma) according to the center proteomic protocols for mass spectrometry. The obtained peptide mass fingerprint (PMF) was used to search through the Swiss-Prot and the National Center for Biotechnology Information (NCBI) non-redundant databases.

### *In silico* cloning of the gene encoding DNA binding protein of the Sf9 cells

Using blastp, block maker and CODEHOP [43,44], a 12-bp DNA binding protein cDNA fragment was amplified from the Sf9 cells using degenerate oligonucleotides as shown below: 5'-TGCACTTCCGGATGccntggttyca-3' (forward) and 5'-GCCCTCGGCCTGCaydatyttytg-3' (reverse) (y for C or T; n for A, C, G or T), which were deduced from a subset of the DNA binding protein. This DNA sequence information was used as a probe to search the *Spodoptera frugiperda* expressed sequence tag (EST) database (http://www.ncbi.nlm.nih.gov/dbEST/) for homologous clones using the BLAST program (http://www.ncbi.nlm.nih.gov/blast/). These ESTs were assembled into contig, and the open reading frames were predicted by ORF finder (http://http//:www.ncbi.nlm.nih.gov/gorf) online software.

### Phylogenetic analysis of the DNA binding protein

The amino acid sequences of the DNA binding protein from Sf9 cells were deduced and nine homologous amino acid sequences were retrieved from GenBank for sequence alignment and phylogenetic analysis. Multiple sequences were aligned with ClutalX, and the phylogenetic analysis of the alignments was conducted using MEGA4. The trees were constructed based on the distances that were obtained using the neighbor-joining method. The reliability of the trees was tested by bootstrapping (1000 replicates) using neighbor-joining and parsimony. The trees were viewed using MEGA4.

### Antibody preparation

The DNA binding protein was expressed in *E. coli* using His-tagged pET-30a (Amersham), and was purified using Ni-NTA agarose (Amersham), according to the manufacturer's instructions. Polyclonal antibodies against the DNA binding were generated by immunizing BABL/c mice.

### Electrophoretic mobility-shift assay and super shift

The following oligonucleotide was used: 5'-CTCATTTATTCCTAGAAATG GTGTAATC-3' (the 12-bp element present in the WSSV *ie1* gene promoter is underlined). The oligonucleotides were end-labeled using the Biotin 3' End DNA Labeling kit (Pierce), according to the manufacturer's instructions. The EMSAs were performed using the Light shift Chemiluminescent EMSA kit (Pierce). A 20 fmol biotin-labeled oligonucleotide was incubated with 10 μg nuclear protein extract from the Sf9 cells for 20 minutes at room temperature in a binding buffer (50 mM Tris, pH 7.4, 2.5 mM EDTA, 0.25 mg/ml poly(dI/dC), 250 mM NaCl, 2.5 mM DTT, 5 mM MgCl_2_ and 20% glycerol). The binding was competed using 5- or 10-fold amounts of the excess unlabeled the oligonucleotides (cold probe). For the super shift, 0.5 or 1 μg of the specific antibody was added after incubation of the nuclear proteins with the biotin-labeled oligonucleotides. The binding complexes were resolved using electrophoresis with 5% native polyacrylamide gel in 0.5 × TBE (0.44 M Tris base, 0.44 M boric acid, and 0.01 M EDTA [pH 8.0]), which was transferred to nylon membranes (Pierce), UV cross linked, and visualized using the Chemiluminescent Nucleic Acid Detection System (Pierce).

### Indirect immunofluorescence assay

The Sf9 cells were washed twice with PBS and were fixed with ice cold anhydrous ethanol for 15 min at 4°C and air dried. The cells were then treated with the DNA binding protein antibody at a 1:300 dilution in PBS for 1 h at 37°C. After washing with PBS, FITC-conjugated goat anti-mouse IgG (Sigma) at a 1:200 dilution was added, and the solution was incubated for 1 h at 37°C. The counter-staining of the nuclei was performed using 4,6-diamidino-2-phenylindole dihydrochloride (DAPI) (Invitrogen). After three washes, the results were observed under a confocal laser scanning microscope (Leica TCS SP2).

## Abbreviations

WSSV: White spot syndrome virus; IE1: Immediate early gene 1; PHB: Prohibitin; HTH: Helix-turn-helix; AcMNPV: Auographa californica multicapsid nuclear polyhedrosis virus; OpMNPV: Orgyia pseudotsugata multicapsid nuclear polyhedrosis virus; AP-1: Activator protein-1; RACE: Rapid amplification of cDNA ends; SP1: Specificity protein-1; TBP: TATA-box-binding protein; EMSA: Electrophoretic mobility-shift assay; MALDI: Matrix assisted laser desorption/ionization; DAPI: 4,6-diamidino-2-phenylindole dihydrochloride; TOF: Time-of-flight; UTR: Untranslated region; Inr: Initiator element.

## Competing interests

The authors declare that they have no competing interests.

## Authors’ contributions

GDM and LY carried out the molecular genetics studies and drafted the manuscript. QW carried out virus preparation and cell culture. YDC participated in the sequence alignment. LY and WL carried out plasmid construction and promoter activity assay. JK conceived the experimental design and participated in revising the manuscript. All authors read and approved the final manuscript.
